# Health behavior and psychological treatment utilization in adults with avoidant/restrictive food intake disorder symptoms

**DOI:** 10.1186/s40337-024-01049-1

**Published:** 2024-06-26

**Authors:** Julia Enya Engelkamp, Andrea Sabrina Hartmann, Katja Petrowski, Benedict Herhaus, Jörg Michael Fegert, Cedric Sachser, Peter Kropp, Britta Müller, Elmar Brähler, Anja Hilbert

**Affiliations:** 1 Department of Psychology, Clinical Psychology and Psychotherapy of Childhood and Adolescence, Konstanz University, Universitätstr. 10, 78464 Konstanz, Germany; 2 Department of Medical Psychology and Medical Sociology, University Medical Centre of the Johannes Gutenberg University Mainz, Langenbeckstr. 1, 55131 Mainz, Germany; 3 Department of Child and Adolescent Psychiatry and Psychotherapy, University of Ulm, Krankenhausweg 3, 89075 Ulm, Germany; 4 Institute of Medical Psychology and Medical Sociology, University Medical Center Rostock, Gehlsheimer Str. 20, 18147 Rostock, Germany; 5 Behavioral Medicine Research Unit, Department of Psychosomatic Medicine and Psychotherapy, Integrated Research and Treatment Center AdiposityDiseases, Leipzig University Medical Center, Stephanstr. 9a, 04103 Leipzig, Germany; 6 Department of Psychosomatic Medicine and Psychotherapy, University Medical Centre of the Johannes Gutenberg University Mainz, Untere Zahlbacher Str. 8, 55131 Mainz, Germany

**Keywords:** Avoidant/Restrictive food intake disorder, Health behavior, Smoking, Alcohol misuse, Treatment utilization

## Abstract

**Background:**

Avoidant/restrictive food intake disorder (ARFID), an eating disorder not associated with weight and shape concerns, results in nutrient or energy deficiencies related with further health consequences and a pronounced need for specialized treatment. These interventions need to be tailored to individual health behavior. However, research about health behavior and treatment utilization in ARFID is scarce, particularly in adults, as ARFID is more common in children despite occurring across the lifespan. One important aspect of health behavior is the individual’s health regulatory focus (i.e., health prevention and health promotion). Additionally, symptoms of eating disorders have generally been associated with various health risk behaviors, such as smoking, drinking, or unhealthy physical (in)activity. Therefore, the present study aimed to investigate health behavior and psychological treatment utilization in adults with symptoms of ARFID.

**Methods:**

A representative adult population sample (*N* = 2415) completed several self-report questionnaires assessing symptoms of eating disorders and health behavior. Differences between groups (symptoms of ARFID vs. no symptoms of ARFID) were tested with analysis of variance, Mann-Whitney-U-tests, and binary logistic regression.

**Results:**

Individuals with symptoms of ARFID (*n* = 20) did not differ in their health regulatory focus, smoking status, physical activity or psychological treatment utilization from individuals without symptoms of ARFID (*n* = 2395). However, they reported higher alcohol misuse than individuals without symptoms of ARFID.

**Conclusion:**

The findings suggest a relevance of further exploration of the relationship between alcohol misuse and ARFID, given the preliminary nature of these results. This exploration could inform treatment strategies for addressing potential comorbid substance misuse. Furthermore, the low psychological treatment utilization in adults with symptoms of ARFID suggest a need for more specialized psychological treatment services, public education about ARFID being an indication for psychological treatment, and further research about treatment barriers.

**Supplementary Information:**

The online version contains supplementary material available at 10.1186/s40337-024-01049-1.

## Abstract (plain language summary)

Avoidant/restrictive food intake disorder (ARFID), an eating disorder not associated with body image or weight concerns, results in nutrient or energy deficiencies related with further health consequences. It is most common in children, but can occur across the lifespan, although there is little research in adults. Therefore, the study investigated if adults with symptoms of ARFID differ from adults without symptoms of ARFID in health behaviors. A total of 2415 adults from a German national population sample completed questionnaires assessing symptoms of ARFID, health regulatory focus (health promotion focus with the aim of improving one’s health and health prevention focus aiming to avoid any deterioration in health), alcohol misuse, smoking behavior, physical activity and psychological treatment utilization. Adults with symptoms of ARFID did not differ from those without symptoms of ARFID in treatment utilization or any of the assessed health behaviors except reporting higher alcohol misuse. We, therefore, suggest to further explore potential alcohol misuse in individuals with ARFID. Furthermore, more research about treatment barriers in ARFID and more specialized psychological treatment services as well as public education about ARFID being an indication for psychological treatment, are needed to address the low psychological treatment utilization.

**Supplementary Information:**

The online version contains supplementary material available at 10.1186/s40337-024-01049-1.

## Introduction

Avoidant restrictive food intake disorder (ARFID) is associated with several health consequences and a high need for specialized treatments [1, 2]. According to the Diagnostic and Statistical Manual of Mental Disorders Fifth Edition (DSM-5; 3) ARFID results in nutrient or energy deficiencies and can be associated with significant weight loss (or failure to achieve expected growth in children), significant nutritional deficiency, dependence on enteral feeding or nutritional supplements, and/or marked interference with psychosocial functioning [3, 4]. Research in children and adults suggests that these health consequences are also linked to further health risk, for instance, decreased bone density [5] and higher rates of medical comorbidities (e.g., endocrine dysfunction and asthma [2]). In contrast to other eating disorders, such as anorexia nervosa, ARFID is not associated with body image and weight concerns [3]. According to the DSM-5, ARFID can manifest in heterogenous presentation, individually or in combination, such as lack of interest in eating, fear of aversive consequences of eating, and food avoidance based on sensory sensitivity to food characteristics [3]. While ARFID can occur across the lifespan, it is more prevalent in younger children, which is in contrast to other eating disorders which occur more frequently later in adolescence [6]. As a consequence of higher childhood prevalence rates, previous research about health behavior (e.g., physical activity) and treatment utilization focuses predominantly on children and adolescents [7–9]. Thus, more research into adult samples is needed.

Children and adults with eating disorders in general exhibit high utilization of mental healthcare services [10, 11]. They utilize health care services more frequently than matched healthy individuals with similar rates across different diagnoses [9, 10]. In ARFID, research about treatment utilization is comparably scarce. This is not surprising, considering the relatively recent introduction of the diagnosis in the DSM-5 [3]. Furthermore, the heterogenous presentation of ARFID and the high need for multidisciplinary treatments raises difficulties for treatment centers to correctly diagnose and adopt treatments to the specific needs of patients with ARFID [1]. Limited research on psychiatric and psychotherapy utilization in ARFID has demonstrated comparable treatment utilization in children with ARFID versus other eating disorders [9]. In adults, there is some evidence that readmission rates for ARFID are lower than those for other eating disorders, but overall psychological treatment utilization in adults is unclear [12].

Beyond the treatment focus on disorder-specific symptoms and the reported health risks, interventions need to consider an individual’s general health behavior (e.g., activity levels) and health motivation to reduce health consequences [13]. One important aspect of health behavior is an individual’s health regulatory focus [13–15]. According to regulatory focus theory, health behavior can be driven by two independent foci, a promotion focus (i.e., approaching desirable outcomes) and a prevention focus (i.e., avoiding undesirable outcomes; [16]). In the context of health behavior, individuals with a high promotion focus seek to advance their current health state, while individuals with a high prevention focus are motivated to maintain their current health and to avoid any deterioration [13–15]. The prevention focus has been associated with lower subjective health and greater somatic and psychological symptoms in German adults (e.g., depression and anxiety [13, 17]), while the promotion focus has been associated with better subjective health and optimism [13]. Additionally, a promotion focus has been associated with health behavior and health behavior intentions such as physical activity, reduction of alcohol consumption and quitting smoking [14, 15].

In contrast, findings for health prevention are mixed. While one study reported a negative association of health prevention to health behavior [14], another yielded a negative correlation only to some health behavior intentions (e.g., to be physically active), but revealed a positive relation to quitting smoking [15]. Interestingly, in adults, a general prevention focus has been associated with emotional eating (i.e., coping with negative emotions), and a promotion focus has been related to external eating (i.e., eating to external cues, such as the smell or appearance of food [18]). Given that low interest in eating – and therefore a low reactivity to external cues such as palatable food – is one of the proposed presentations of ARFID [19], the promotion focus might be relatively low in individuals with ARFID. However, evidence about the regulatory focus motivating health behavior in ARFID is lacking across all ages and different presentations of ARFID. Importantly, most behaviors are assumed to be not inherently promotive or preventive in general, and considering an individual’s regulatory focus in behavioral intervention (e.g., by matching the framing of a health goal to the respective focus) can enhance health behavior intentions [20]. Hence, knowledge about promotion and prevention focus in individuals with ARFID could potentially benefit health interventions in ARFID to reduce health risks.

Apart from general health behavioral tendencies, symptoms of eating disorders have generally been associated with various health risk behaviors. First, adults with eating disorders are at higher risk for smoking [21, 22]. In particular, restrictive eating disorders have been associated with smoking status [23]. Second, in adults, eating disorders have been shown to co-occur with alcohol misuse and dependence [22, 24]. In ARFID, relatively little is known about substance misuse behaviors, although a case study in an adult with ARFID suggests comorbidities with alcohol substance disorder [25]. Third, eating disorders have been associated with inadequate physical activity in adults, depending on the diagnosis, with some cases involving too much physical activity (e.g., compensatory behavior in anorexia nervosa [26]) and others involving insufficient physical activity [27]. Interestingly, picky eating has been associated with physical inactivity in young children [7, 8], but if and how this association also occurs in adulthood is unknown.

Despite its severe health consequences, information about health behavior in adults with ARFID is currently lacking. It is unclear whether smoking and alcohol misuse are elevated, similar to other eating disorders [22], and whether physical inactivity associated with picky eating in childhood [7] extends into adulthood. Therefore, the aim of the present study was to investigate health behavior and psychological treatment utilization in adults with symptoms of ARFID. In line with previous research on eating disorders, we hypothesized that adults with symptoms of ARFID would more likely be physically inactive, smoke, and misuse alcohol than those without symptoms of ARFID. Furthermore, given the positive association of a promotion focus with health behaviors which are less common in eating disorders [14, 15], as well as its association with external eating – an eating behavior not strongly present in some individuals with ARFID – we hypothesized a lower health promotion focus in adults with symptoms of ARFID than those without. Similarly, based on the association of the prevention focus with lower subjective health and greater psychological symptoms [13, 17], we hypothesized that adults with symptoms of ARFID would show higher health prevention focus than those without symptoms of ARFID.

Additionally, adults with symptoms of ARFID were hypothesized to be more likely to have sought psychological treatment (not necessarily ARFID-related) than those without symptoms of ARFID.

## Method

### Participants and design

USUMA (Berlin, Germany), an independent of market, opinion, and social research agency collected the data on a representative German sample aged ≥14 years and fluent in German as inclusion criteria. The recruitment period comprised September to November 2016. The sampling procedure used sampling regions from 258 defined point regions in Germany, a random route procedure to select households, and a Kish selection grid to choose individuals within these households. The detailed procedure has been described elsewhere [28]. *N* = 2510 participants out of 4902 selected households participated. Reasons for nonparticipation of households included refusal (*n* = 738), households were unreachable (*n* = 723), or did not met the inclusion criteria (*n* = 15); individuals targeted in the households refused participation (*n* = 715), were unreachable within four contact attempts (*n* = 111) or had other/unknown reasons (*n* = 81) [28]. For the present analysis, only adults were included (exclusion of *n* = 86 individuals due to age < 18 years). Nine individuals were excluded because of missing items critical for determining symptoms of ARFID. *N* = 45 individuals exhibited symptoms of eating disorders (ED) other than ARFID (EDE-Q8 score > 97% percentile [29]) and were therefore excluded. Additionally, for *n* = 12 data on symptoms of eating disorders was not available and they were therefore also excluded, resulting in a final sample of *N* = 2358.

### Procedure

Trained research assistants visited participants at home to provide information about the procedure, obtain informed consent, and supervise the self-report assessment. Participants did not receive an incentive for participation. The procedure followed the ethical guidelines of the International Code of Marketing and Social Research Practice by the International Chamber of Commerce and the European Society for Opinion and Marketing Research, and ethical approval was obtained from the Ethics Committee of the University of Leipzig [28]. Additional information about the quality control of the data assessment is provided in the Additional file 1.

### Measures

#### Eating disorders in youth-questionnaire (EDY-Q)

The EDY-Q [30] measures self-reported restricted eating disturbances. This questionnaire consists of 14 items rated on a 7-point Likert scale (0 = “never” to 6 = “always”), with 12 items necessary to measure symptoms of ARFID.

Low interest in food, fear of aversive consequences, sensory sensitivity and problems with underweight are each assessed with one item. Two items measure shape and weight concerns serving as exclusion criteria for symptoms of ARFID. The EDY-Q only assesses problems with underweight as a consequence of restrictive eating, therefore other potential consequences (e.g., psychosocial impairments) that might indicate the presence of symptoms of ARFID in the absence of significant weight loss could not be considered.

Thus, ratings ≥ 4 (= often) on at least one of the three inclusion items measuring food restriction, ratings ≥ 4 (often) for weight problems, and ratings of 2 (= less than sometimes) or lower on the exclusion items suggested symptoms of ARFID. The EDY-Q has been previously validated in the present adult population data, showing satisfactory discriminant and divergent validity, and internal consistency Cronbach’s α = 0.67 [28]. However, for the present analyses the total score was not used, only the individual items addressing the symptoms of ARFID were employed.

#### Health regulatory focus scale (HRFS)

The Health Regulatory Focus Scale (HRFS) is an eight-item self-report questionnaire assessing health promotion and prevention focus. The translated German-language version from Schmalbach et al. (2017) was used [13]. All items are rated on a 7-point Likert scale (1 = “strongly disagree” to 7 = “strongly agree”). The average of the items for the health promotion subscale (5 items) and health prevention subscale (3 items) were used as an aggregated score in the analysis. The internal consistency in the present data set was acceptable to high, with α = 0.92 for health promotion and α = 0.76 for health prevention.

#### Cut-down annoyed guilty eyeopener (CAGE)

The Cut-down Annoyed Guilty Eyeopener (CAGE [31]), is a self-report questionnaire assessing alcohol misuse. It consists of 4 items with dichotomous answer options (1 = “yes”; 0 = “no”). The items measure perceived need to cut down (item 1), feeling bad or guilty (item 2) or having been criticized (item 3) about drinking, or using alcohol as an eyeopener (item 4). The total score was calculated as an aggregated score. Additionally, the recommended cut-off value of ≥ 2 was used to identify individuals with problematic alcohol misuse [32]. The internal consistency in the present sample was acceptable, with α = 0.79.

#### Smoking status

The single-item self-report question “Do you smoke?” was used to measure smoking status via the dichotomous answer option 1 = “yes” versus 0 = “no”.

#### Physical inactivity

The single-item self-report question “Do you exercise regularly (i.e., on average at least 2–3 times a week for 30 min or longer)?” was used to measure physical inactivity via the dichotomous answer option 0 = ”yes” versus 1 = “no”.

#### Psychological treatment utilization

Two items measured the utilization of psychological (defined as psychosomatic/psychiatric/psychotherapeutic) treatment, one for outpatient and one for inpatient settings. Participants rated the frequency of received treatments on 5-point ordinal scales (1 = “never”, 2 = “1 to 3 times”, 3 = “4 to 6 times”, 4 = “7 to 9 times”, 5 = “more than 10 times”). For the analysis, psychological treatment had to be recoded in dichotomous answer options as either psychological treatment received (coded as 1) or as not received (coded as 0), due to an absence of ratings across all categories.

#### Eating disorder examination-questionnaire-8 (EDE-Q8)

To control for eating disorder symptomology other than ARFID, global eating disorder psychopathology was assessed via the EDE-Q8 to identify individuals with symptoms of other eating disorders beyond ARFID. This short form of the EDE-Q [33, 34] consists of eight items rated on a 7-point Likert scale (0 = “not present” to 6 = “present every day/in extreme form”) measuring restrained eating and eating, weight, and shape concern. Individuals with a mean score above the 97th percentile [29] were categorized as having symptoms of an eating disorder. Internal consistency in the present data set was high, α = 0.91.

#### Patient health questionnaire-4 (PHQ-4)

The PHQ-4 self-report questionnaire consists of four items, two items assessing symptoms of depression and anxiety, respectively. All items are rated on a 4-point Likert scale (0 = “not at all” to 3 = “nearly every day”). The aggregated sum score was used for the analyses. The questionnaire has been validated in population studies [35] showing good construct validity. Internal consistency in the present sample was good, α = 0.88.

#### Sociodemographic and clinical variables

Participants self-reported their age, gender, and ethnicity. Subsequently, individuals were divided into three age groups (18–39 years, 40–59 years, ≥ 60 years). Additionally, the Body Mass Index (BMI; kg/m^2^) was derived from self-reported weight and height and all individuals were divided into weight status groups (underweight, < 18.5 kg/m^2^, normal weight, 18.5–24.9 kg/m^2^, overweight, 25.0–29.9 kg/m^2^, obesity, ≥ 30 kg/m^2^).

### Statistical analysis

All analyses were preregistered at OSF [36]. For statistical reasons, a few deviations from the original planned and preregistered analysis were necessary and are highlighted in the method and results sections. Individuals with and without symptoms of ARFID were identified based on the described items in the EDY-Q. Similarly, individuals with symptoms of other eating disorders were categorized based on the EDE-Q8. χ^2^ tests were applied for group comparisons among age groups, gender, and weight status. The Shapiro-Wilks normality test was conducted to test for normality in the group of adults with symptoms of ARFID. Given the large sample size in the group of adults without symptoms of ARFID, the recommended measures for normality testing in large sample sizes (absolute skewness < 2 and kurtosis level < 4) [37] were applied. Differences between groups (symptoms of ARFID vs. no symptoms of ARFID) in health promotion, health prevention, and alcohol misuse were tested with analysis of variance (ANOVA), and in case of normality violation with Mann-Whitney-U-tests. For dichotomous variables (binary), logistic regression was conducted to assess group differences in smoking, physical activity, and psychological treatment utilization. Individuals that have not filled out at least 80% of the items per scale were excluded listwise from the analysis of the respective scale and missing at random was assumed. All analyses were repeated with individuals with eating disorder symptoms other than ARFID included in the control group (see Additional file 2).

## Results

### Sample characteristics

The complete sample consisted of *N* = 2358 individuals with a mean age of 49.56 years (*SD* = 17.48). Approximately half of the sample identified as female (*n* = 1251, 53.05%) and the majority were of German nationality (*n* = 2281; 96.73%). The average BMI was 25.79 kg/m^2^ (*SD* = 4.54) and *n* = 1820 individuals (77.18%) had less than 12 years of education. Individuals with symptoms of ARFID (*n* = 20, 0.85%) did not differ from individuals without symptoms of ARFID in age or gender (see Additional file 1), but in weight status. Furthermore, individuals with symptoms of ARFID had higher depression and anxiety levels compared to individuals without symptoms of ARFID *F*(1, 2350) = 9.47, *p* = .002; *η*^*2* =^ 0.004.

### Health regulatory focus

Adults with symptoms of ARFID did not differ from adults without symptoms of ARFID in health promotion, or prevention focus as displayed in Table 1.

**Table 1:** One-way analyses of variance of health promotion and health prevention focus in individuals with and without ARFID

Measure	With ARFID symptoms (n = 20)		Without ARFID symptoms (n = 2338)		*df*	*F*	*p*	η^2^
	*M*	*SD*	*M*	*SD*				
Health promotion	4.24	1.42	4.25^1^	1.43	(1, 2352)	0.001	.98	.00
Health prevention	3.85	1.57	3.69	1.40	(1, 2346)	0.25	.62	.00

Note: Total *N* = 2358. Health promotion focus and health prevention focus assessed via the Health Regulatory Focus Scale. ARFID: Avoidant/Restrictive Food Intake Disorder; M: Mean; SD: Standard Deviation;

^1^Missing values: *N* = 4 individuals without symptoms of ARFID had missing values for health promotion, *n* = 10 individuals without symptoms of ARFID had missing values for health prevention focus

### Health behaviors and psychological treatment utilization

Reported in Table 2 is the prevalence of health behaviors in individuals with and without symptoms of ARFID. Regression models suggested no significant difference in alcohol misuse, smoking status, or physical inactivity (Table 3) with odd ratios (OR) ranging from 0.5 to 2.3. Furthermore, individuals with and without symptoms of ARFID did not differ significantly in outpatient or inpatient psychological treatment utilization (Table 3). The non-parametric Mann-Whitney-U-test revealed significantly higher alcohol misuse in individuals with versus those without symptoms of ARFID (*U* = 27034.50, *p* = .03). However, the higher misuse in individuals with symptoms of ARFID (*OR* = 2.3), did not significantly predict alcohol misuse vs. non-misuse in a binary regression model (*p* = .11), suggesting no significant difference in alcohol (non)-misuse status (Table 3). All analyses revealed similar results when individuals with other eating disorders (*n* = 48) were not excluded from the overall data set (see Additional file 2 for detailed results).

**Table 2:** Health behavior in individuals with/without symptoms of ARFID

	ARFID symptoms		Without ARFID symptoms	
	*n*	%	*n*	%
Alcohol				
No misuse (ref)	15	75.0	1963	87.4
Misuse	5	25.0	282	12.6
Smoking status				
Non-smoker (ref)	10	50.0	1527	65.7
Smoker	10	50.0	798	34.3
Physical inactivity				
Active (ref)	11	55.0	850	38.2
Inactive	9	45.0	1373	61.8
Psychological treatment outpatient				
No treatment (ref)	17	85.0	2127	91.6
Utilized treatment	3	15.0	194	8.4
Psychological treatment inpatient				
No treatment (ref)	19	95.0	2231	96.0
Utilized treatment	1	5.0	94	4.0

Note: Symptoms of ARFID assessed via the Eating Disorders in Youth-Questionnaire. *N* = 93 adults for alcohol, *n* = 13 for smoking status, *n* = 115 for physical inactivity, *n* = 13 psychological treatment outpatient, *n* = 13 for psychological treatment inpatient, individuals without symptoms of ARFID were excluded from the analysis respectively, due to missing data. ARFID: Avoidant/Restrictive Food Intake Disorder, ED: Eating Disorder, Ref: Reference group

**Table 3:** Health behavior variations by presence of ARFID symptoms

Health behavior	Test statistics					
				95% CI for odds ratio		
	B	SE	*p*	LL	OR	UL
Alcohol						
No misuse (ref)				ref	ref	ref
Misuse	0.84	0.52	0.11	0.84	2.32	6.43
Smoking status						
Non-smoker (ref)				ref	ref	ref
Smoker	0.65	0.45	0.15	0.79	1.91	4.62
Physical inactivity						
Active (ref)				ref	ref	ref
Inactive	-0.68	0.45	0.13	0.21	0.51	1.23
Psychological treatment outpatient						
No treatment (ref)				ref	ref	ref
Utilized treatment	0.66	0.63	0.30	0.56	1.94	6.66
Psychological treatment inpatient						
No treatment (ref)				ref	ref	ref
Utilized treatment	0.22	1.03	0.83	0.17	1.25	9.43

Note: Differences in health behavior from binary logistic regression models in symptoms of ARFID (with/without) assessed via the Eating Disorders in Youth-Questionnaire, excluding individuals with other eating disorders (*n* = 45). *N* = 99 adults for alcohol, *n* = 15 for smoking status, *n* = 89 for physical inactivity, *n* = 20 psychological treatment outpatient, *n* = 19 for psychological treatment inpatient, individuals without symptoms of ARFID were excluded from the analysis respectively, due to missing data. ARFID: Avoidant/Restrictive Food Intake Disorder; B unstandardized regression coefficient, SE: Standard Error CI: Confidence Interval; Ref: Reference group; OR: Odds Ratio; LL: Lower Level; UL: Upper Level

## Discussion

This study aimed to investigate health behaviors and psychological treatment utilization in adults with versus those without symptoms of ARFID in a representative adult German population survey. Findings did not yield differences between adults with and those without symptoms of ARFID in their health regulatory focus, smoking status, or physical inactivity. However, those with symptoms of ARFID reported significantly higher alcohol misuse. Rates of psychological treatment utilization were not significantly different between adults with and without symptoms of ARFID.

The present nonsignificant findings of differences in health promotion are in line with previous population-based studies suggesting low to non-existent associations for health promotion focus with mental health [17]. The finding, however, is surprising provided that previous research has shown an association between general not health-related promotion focus with external eating [18]. Given the fact that some individuals with ARFID show low interest in food [3] and thus, might be less likely to engage in external eating, one might have expected low promotion focus in individuals with ARFID. The nonsignificant difference in the promotion focus between groups in our study suggests that the above-mentioned finding on associations with external eating might not transfer from a general to a health context-specific regulatory focus. This is supported by research showing that health regulatory focus, but not general promotion or prevention focus predict health behaviors (e.g., dentist visits, use of prescription drugs; [14]). However, the results should be interpreted with caution due to the small effect sizes of the analysis and the small number of adults with symptoms of ARFID, which also did not allow to investigate for group differences among the presentations of ARFID and their association with external eating. Additionally, the findings are limited to individuals with symptoms of ARFID who reported problems with underweight. Further research investigating the relationship of symptoms of ARFID, external eating tendencies and health promotion is needed.

In contrast to previous studies revealing an association of general psychopathology with high health prevention focus [13, 17], adults with symptoms of ARFID did not show a significant difference in prevention focus compared to those without symptoms of ARFID. The findings, however, should be interpreted considering the afore-mentioned limitations of the analysis, including the unequal group sizes, the low number of individuals with symptoms of ARFID and the inclusion of only individuals with symptoms of ARFID reporting problems with underweight. Additionally, the lack of significant findings might be due to the population-based sample, as previous studies indicated lower correlations between mental health and health prevention focus in population-based studies [17] than in convenience samples [13]. Also, greater correlations have been found in younger as opposed to older samples [13, 17]. Thus, the size of the association might depend on personal characteristics (e.g., age).

Regarding specific health behaviors, symptoms of ARFID did not predict physical activity or smoking status. Interestingly, in the present sample, the regression analysis for being physical inactive, revealed a lower odds ratio to be inactive for individuals with symptoms of ARFID than without. These findings contrast with studies in children associating picky eating with physical inactivity reported by parents [7, 8]. However, given the difference in both sample age and report (self- versus other-rating), compatibility of these studies with our findings can be called into question. Additionally, our binary assessment did not allow for comparisons of a broader range of the activity level beyond the amount of physical activity assessed (i.e., two to three times per week for 30 min). Moreover, as previously pointed out, the present study focussed only on individuals with symptoms of ARFID who had a low to normal BMI and reported problems with underweight. Further research in ARFID is needed to investigate differences in physical activity for different weight groups. Furthermore, although the presence of symptoms of ARFID did not significantly predict smoking status, smoking status was generally quite high in individuals with symptoms of ARFID (50%) and descriptively the odds were nearly twice as high in individuals with symptoms of ARFID to be a smoker compared to individuals without. Since smoking can reduce sensory sensitivity and alter taste [38, 39], it would be interesting to further investigate smoking motivation in individuals with ARFID presenting with food avoidance based on sensory characteristics of food (e.g., as a potential coping mechanism for food sensory sensitivity).

Individuals with symptoms of ARFID exhibited higher alcohol misuse than individuals without symptoms of ARFID. This result is in line with research reporting co-occurrence of alcohol use and eating disorders [22, 23]. Thus, individuals with ARFID might be more vulnerable to unhealthy drinking, which is also consistent with findings from an adult case study [25]. However, symptoms of ARFID alone did not significantly predict alcohol misuse, which could be attributed to the very small effect sizes in the present analyses, but odds ratio indicated more than twice as high odds for individuals with symptoms of ARFID to misuse alcohol compared to individuals without. More research with formal diagnoses of alcohol misuse is necessary to confirm the findings and to further explore the relationship between ARFID and alcohol misuse.

A second aim of the present study was to investigate psychological treatment utilization in ARFID. The vast majority of individuals with symptoms of ARFID in our sample were untreated, and the number of out- and inpatient psychological treatments did not differ significantly for individuals with versus those without symptoms of ARFID. This is in line with the fact that overall psychological treatment utilization in the present sample was comparable to a previous psychological treatment utilization study reporting that 9.7% of the German population utilized psychological treatment at least once in the assessed 12-month period [40]. However, these findings should be interpreted with care, due to the absence of a formal ARFID diagnosis or inclusion of other mental disorders or medical conditions. Additionally, due to the low number of adults with symptoms of ARFID who received treatment and absence of individuals with more than three treatments, we could not investigate group differences in the frequency of treatments. However, given that most of our participants with symptoms of ARFID never received psychological treatment, it could be argued that they either did not consider their symptoms to be an indication for psychological treatment or did not find specialized treatment programs.

Several strengths and limitations should be considered when interpreting our results. The large representative sample provided a unique opportunity to assess health behavior and symptoms of ARFID balanced for gender and age. The response rate of contacted individuals of 51% is comparable to standard response rates of surveys in Germany [41, 42]. In comparison to data of the Federal Statistical Office 2016, we had slightly more adults younger than 60 years (69.1% compared to 66.9%), and slightly more females (53.1% compared to 50.7%) in the data set. Therefore, the generalizability of the present findings is slightly limited. Furthermore, in comparison to objective assessment, self-reported weight and height indicated lower prevalence of obesity in the present sample (14.7% in women and 12.5% in men) compared to studies applying objective measures (23.9% in women and 23.3% in men; [43]), but rather similar for reported underweight (1.3% in women and 0.7% in men compared to 2.3% in women and 0.7% in men [43]).

Moreover, the focus on adults with symptoms of ARFID extends research findings predominantly focusing on children with ARFID. However, the present findings are limited by the self-reported assessment of health behaviors and symptoms of ARFID without a confirmed clinical diagnosis. Additionally, the present study focused only on individuals with symptoms of ARFID who reported problems with underweight. This is due to the fact that this is the only consequence assessed with the EDY-Q. The EDY-Q was developed prior to the release of the DSM-5. While it has items to assess the different presentations of the disorder (low interest in food, fear of aversive consequences, sensory sensitivity), it does not yet reflect the different manifestations (e.g., social impairment) of the three presentations beyond underweight. Thus, the applicability of the present findings to individuals with symptoms of ARFID exhibiting other common impairments remains to be shown. Furthermore, we did not evaluate the potential impact of other symptoms of mental disorders on health behavior, as this was beyond the scope of the paper, which may have impacted the self-reported health behaviors. Overall, the present analyses were limited by the unequal group sizes, the small group of individuals with symptoms of ARFID in the sample, the focus on individuals with symptoms of ARFID reporting problem with underweight and the use of a dichotomous health behavior classification (e.g., treatment utilization). Thus, findings from – in particular - the regression analyses need to be interpreted with care. It was also not possible to investigate the association of health behaviors with different presentations of ARFID, due to the small number of individuals with symptoms of ARFID. Their connection to sensory sensitivity or lack of interest in food needs to be further investigated.

The present findings have implications for research and clinical practice for ARFID. If increased alcohol misuses and smoking in adults with symptoms of ARFID are replicated, screening for potential substance use disorders and related health behaviors could inform clinical interventions. Further research is needed to investigate potential linkages between smoking behavior and restrictive eating in ARFID (e.g., to decrease sensory sensitivity through smoking [44]). Furthermore, since smoking and physical inactivity are associated with medical comorbidities of ARFID, such as asthma [2, 45, 46], further investigations are necessary for shedding more light on the interplay of comorbid medical conditions, health behaviors, and symptoms of ARFID. The low endorsement of treatment utilization by individuals with symptoms of ARFID implies a need for further investigation into causes of low treatment utilization and treatment barriers.

Overall, the present study first provided insight into health regulatory focus, prevalence of alcohol misuse, smoking, physical inactivity and psychological treatment utilization in adults with symptoms of ARFID. The findings suggest that adults with symptoms of ARFID do not differ from those without symptoms of ARFID with regard to health regulatory focus or specific health behaviors with the exception of alcohol misuse. The rather low utilization of psychological treatments found in the present sample, despite potentially associated severe health consequences, underline the twin needs for raising public awareness about the condition and for improving the clinical care of individuals with ARFID in Germany.

### Electronic supplementary material

Below is the link to the electronic supplementary material.

Supplementary Material 1

Supplementary Material 2

## Data Availability

The datasets analyzed during the current study are not publicly available related to the ethical approval, but can be obtained from the last author (AH) upon reasonable request.

## Abbreviations

ANOVA: Analysis of variance
ARFID: Avoidant/restrictive food intake disorder
B: Regression coefficient
BMI: Body Mass Index
CAGE: Cut-down Annoyed Guilty Eyeopener
CI: Confidence Interval
EDY-Q: Eating Disorders in Youth-Questionnaire
HRFS: Health Regulatory Focus Scale
LL: Lower Level
OR: Odds Ratio
Ref: Reference group
SE: Standard error
UL: Upper Level
