# Supplementary material for: Health behavior and psychological treatment utilization in adults with avoidant/restrictive food intake disorder symptoms
Source: J Eat Disord. 2024 Jun 26;12:88. doi: 10.1186/s40337-024-01049-1 (PMC11200871; doi:10.1186/s40337-024-01049-1)
Supplement: Supplementary file 1 — Supplementary Material 1 [file 40337_2024_1049_MOESM1_ESM.docx]

**Additional file 1**

**Health Behavior and Psychological Treatment Utilization in Adults with Avoidant/Restrictive Food Intake Disorder Symptoms**

**By J. E. Engelkamp, A. S. Hartmann, K. Petrowski, B. Herhaus, J. M. Fegert, C. Sachser, P. Kropp, B. Müller, E. Brähler, A. Hilbert**

**Table 1**

*Socio-demographic variations in individuals with and without symptoms of ARFID*

| Sample characteristics | ARFID  symptoms (*n* = 20) | | Without ARFID  symptoms (*n* = 2338) | | Test statistics | | | |
| --- | --- | --- | --- | --- | --- | --- | --- | --- |
|  | *n* | % | *n* | % | χ^2^ | df | p | Cramer’s V |
| Gender |  |  |  |  | .08 | 1 | .83 | .01 |
| Female | 10 | 50.0 | 1241 | 53.1 |  |  |  |  |
| Male | 10 | 50.0 | 1097 | 46.9 |  |  |  |  |
| Age |  |  |  |  | 1.19 | 2 | .55 | .02 |
| 18–39 years | 8 | 40.0 | 757 | 32.4 |  |  |  |  |
| 40–59 years | 8 | 40.0 | 857 | 36.7 |  |  |  |  |
| ≥ 60 years | 4 | 20.0 | 724 | 31.0 |  |  |  |  |
| Weight status^1^ |  |  |  |  | 55.53 | 3 | <.001 | .16 |
| Underweight, < 18.5 kg/m^2^ | 3 | 15.8 | 21 | 0.9 |  |  |  |  |
| Normal weight, 18.5 – 24.9 kg/m^2^ | 16 | 84.2 | 1088 | 47.3 |  |  |  |  |
| Overweight, 25.0 – 29.9 kg/m^2^ | 0 | 0.0 | 874 | 38.0 |  |  |  |  |
| Obesity, ≥ 30 kg/m^2^ | 0 | 0.0 | 317 | 13.8 |  |  |  |  |

*Note:* Total *N =* 2358. Symptoms of ARFID assessed via the Eating Disorders in Youth-Questionnaire. ARFID: Avoidant/Restrictive Food Intake Disorder. ^1^Weight status was derived from self-reported weight and height. Missing values: *N* = 1 adult with symptoms of ARFID had missing values and *n* = 38 adults without symptoms of ARFID had missing values in self-reported weight and height and were therefore excluded from the weight group analysis.

**Quality control**

All assessments were supervised by an assessor who visited the participants at home. The assessments were controlled for completeness and correctness (of address and assessed individual). Additionally, assessors were controlled with a random selection of 43.7% of conducted interviews by sending a postcard to the address with control questions (duration, topic, and perceived criteria for selecting the participants) of which 53.5% were send back to the institute, all of them confirming the assessment. The completeness of the questionnaires, were checked by USUMA. The questionnaires were scanned by a data entry software and error search programs checked the plausibility and formal integrity. All open questions as well as hand written numbers were additionally compared by trained employees. During data cleaning, all abnormalities in the data set were also checked with the original questionnaires.
