# Supplementary material for: Health behavior and psychological treatment utilization in adults with avoidant/restrictive food intake disorder symptoms
Source: J Eat Disord. 2024 Jun 26;12:88. doi: 10.1186/s40337-024-01049-1 (PMC11200871; doi:10.1186/s40337-024-01049-1)
Supplement: Supplementary file 2 — Supplementary Material 2 [file 40337_2024_1049_MOESM2_ESM.docx]

**Additional file 2**

**Health Behavior and Psychological Treatment Utilization in Adults with Avoidant/Restrictive Food Intake Disorder Symptoms**

**By J. E. Engelkamp, A. S. Hartmann, K. Petrowski, B. Herhaus, J. M. Fegert, C. Sachser, P. Kropp, B. Müller, E. Brähler, A. Hilbert**

**Supplemental Results and Tables**

Results for individuals with other eating disorders (ED) included

Table 1b One-way analyses of variance of health promotion and health prevention focus in individuals with and without ARFID

Table 2b Health behavior in individuals with and without symptoms of ARFID

Table 3b Health behavior variations by presence of ARFID symptoms

**Results for individuals with other ED included**

When adults with other eating disorders *n* = 45 were not excluded from the analysis, results were similar to the main analyses (see Table 1b -3b).

**Table 1b**

*One-way analyses of variance of health promotion and health prevention focus in individuals with and without ARFID*

| Measure | With ARFID symptoms  (n=20) | | Without ARFID symptoms  (n=2395) | | *df* | *F* | *p* | η^2^ |
| --- | --- | --- | --- | --- | --- | --- | --- | --- |
|  | *M* | *SD* | *M* | *SD* |  |  |  |  |
| Health promotion | 4.24 | 1.42 | 4.25^1^ | 1.44 | (1, 2409) | <0.001 | .98 | .00 |
| Health prevention | 3.85 | 1.57 | 3.71 | 1.41 | (1, 2403) | 0.21 | .65 | .00 |

*Note:* Total *N =* 2415. Health Promotion Focus and Health Prevention Focus assessed via the Health Regulatory Focus Scale. ARFID: Avoidant/Restrictive Food Intake Disorder; M: Mean; SD: Standard Deviation;

^1^Missing values: *N* = 4 individuals without symptoms of ARFID had missing values for health promotion, *n* = 10 individuals without symptoms of ARFID had missing values for health prevention focus.

**Table 2b**

*Health behavior in individuals with and without symptoms of ARFID*

|  | ARFID  Symptoms | | Without ARFID Symptoms | |
| --- | --- | --- | --- | --- |
|  | *n* | % | *n* | % |
| Alcohol |  |  |  |  |
| No misuse (ref) | 15 | 75.0 | 2005 | 87.3 |
| Misuse | 5 | 25.0 | 291 | 12.7 |
| Smoking status |  |  |  |  |
| Non-smoker (ref) | 10 | 50.0 | 1560 | 65.5 |
| Smoker | 10 | 50.0 | 820 | 34.5 |
| Physical inactivity |  |  |  |  |
| Active (ref) | 11 | 55.0 | 869 | 38.1 |
| Inactive | 9 | 45.0 | 1410 | 61.9 |
| Psychological treatment outpatient |  |  |  |  |
| No treatment (ref) | 17 | 85.0 | 2166 | 91.2 |
| Utilized treatment | 3 | 15.0 | 209 | 8.8 |
| Psychological treatment inpatient |  |  |  |  |
| No treatment (ref) | 19 | 95.0 | 2278 | 95.9 |
| Utilized treatment | 1 | 5.0 | 98 | 4.1 |

Notes: Symptoms of ARFID assessed via the Eating Disorders in Youth-Questionnaire. *N* = 99 adults for alcohol, *n* = 15 for smoking status, *n* = 116 for physical inactivity, *n* = 20 psychological treatment outpatient, *n* = 19 for psychological treatment inpatient, individuals without symptoms of ARFID were excluded from the analysis respectively, due to missing data. ARFID: Avoidant/Restrictive Food Intake Disorder; Ref: Reference group.

**Table 3b**

*Health behavior variations by presence of ARFID symptoms*

| Health behavior | Test Statistics | | | | | |
| --- | --- | --- | --- | --- | --- | --- |
|  |  | |  | 95% CI for odds ratio | | |
|  | *B* | *SE* | *p* | *LL* | *OR* | *UL* |
| Alcohol |  |  |  |  |  |  |
| No misuse (ref) |  |  |  | ref | ref | ref |
| Misuse | 0.83 | 0.52 | .11 | 0.83 | 2.30 | 6.37 |
| Smoking status |  |  |  |  |  |  |
| Non-smoker (ref) |  |  |  | ref | ref | ref |
| Smoker | 0.64 | 0.45 | .15 | 0.79 | 1.90 | 4.59 |
| Physical inactivity |  |  |  |  |  |  |
| Active (ref) |  |  |  | ref | ref | ref |
| Inactive | -0.69 | 0.45 | .13 | 0.21 | 0.50 | 1.2 |
| Psychological treatment outpatient |  |  |  |  |  |  |
| No treatment (ref) |  |  |  | ref | ref | ref |
| Utilized treatment | 0.60 | 0.63 | .34 | 0.53 | 1.83 | 6.30 |
| Psychological treatment inpatient |  |  |  |  |  |  |
| No treatment (ref) |  |  |  | ref | ref | ref |
| Utilized treatment | 0.20 | 1.03 | .85 | 0.16 | 1.22 | 9.23 |

*Note:* Differences in health behavior from binary logistic regression models in symptoms of ARFID (with/without) assessed via the EDY-Q. All analyses revealed similar results when individuals with other eating disorders (*n* = 48) were excluded from the overall data set (see Additional file 1). *N* = 99 adults for alcohol, *n* = 15 for smoking status, *n* = 116 for physical inactivity, *n* = 20 psychological treatment outpatient, *n* = 19 for psychological treatment inpatient, individuals without symptoms of ARFID were excluded from the analysis respectively, due to missing data. ARFID: Avoidant/Restrictive Food Intake Disorder; B unstandardized regression coefficient, SE: Standard Error CI: Confidence Interval; Ref: Reference group; OR: Odds Ratio; LL: Lower Level; UL: Upper Level.
